# Evaluation of ChatGPT as a diagnostic tool for medical learners and clinicians

**DOI:** 10.1371/journal.pone.0307383

**Published:** 2024-07-31

**Authors:** Ali Hadi, Edward Tran, Branavan Nagarajan, Amrit Kirpalani

**Affiliations:** 1 Department of Paediatrics, Schulich School of Medicine and Dentistry, Western University, London, Ontario, Canada; 2 Division of Nephrology, Children’s Hospital, London Health Sciences Centre, London, Ontario, Canada; Hamad Medical Corporation, QATAR

## Abstract

**Background:**

ChatGPT is a large language model (LLM) trained on over 400 billion words from books, articles, and websites. Its extensive training draws from a large database of information, making it valuable as a diagnostic aid. Moreover, its capacity to comprehend and generate human language allows medical trainees to interact with it, enhancing its appeal as an educational resource. This study aims to investigate ChatGPT’s diagnostic accuracy and utility in medical education.

**Methods:**

150 Medscape case challenges (September 2021 to January 2023) were inputted into ChatGPT. The primary outcome was the number (%) of cases for which the answer given was correct. Secondary outcomes included diagnostic accuracy, cognitive load, and quality of medical information. A qualitative content analysis was also conducted to assess its responses.

**Results:**

ChatGPT answered 49% (74/150) cases correctly. It had an overall accuracy of 74%, a precision of 48.67%, sensitivity of 48.67%, specificity of 82.89%, and an AUC of 0.66. Most answers were considered low cognitive load 51% (77/150) and most answers were complete and relevant 52% (78/150).

**Discussion:**

ChatGPT in its current form is not accurate as a diagnostic tool. ChatGPT does not necessarily give factual correctness, despite the vast amount of information it was trained on. Based on our qualitative analysis, ChatGPT struggles with the interpretation of laboratory values, imaging results, and may overlook key information relevant to the diagnosis. However, it still offers utility as an educational tool. ChatGPT was generally correct in ruling out a specific differential diagnosis and providing reasonable next diagnostic steps. Additionally, answers were easy to understand, showcasing a potential benefit in simplifying complex concepts for medical learners. Our results should guide future research into harnessing ChatGPT’s potential educational benefits, such as simplifying medical concepts and offering guidance on differential diagnoses and next steps.

## Introduction

Artificial Intelligence (AI) refers to computer systems that can perform tasks that require human intelligence, such as visual perception, decision-making, and language understanding [1]. Natural Language Processing (NLP), a crucial field in AI, focuses on the interaction between human language and computer systems [2]. NLP algorithms are capable of analyzing and generating human language, making them valuable tools in various sectors, including healthcare [2].

In the healthcare sector, NLP can be applied in several ways, such as in clinical documentation, coding and billing, monitoring drug safety, and keeping track of patients [3–5]. Large Language Models (LLMs) are a type of NLP model that can perform various language tasks, such as text completion, summarization, translation, and question-answering [6]. LLMs are trained on massive amounts of text data and can generate human-like responses to natural language queries [6, 7].

ChatGPT is a Large Language Model (LLM) developed by OpenAI, capable of performing a diverse array of natural language tasks [8]. At the moment, ChatGPT is arguably the most well-known, commercially available LLM. Its widespread accessibility appeals to a broad audience, including medical trainees and physicians, who are likely to be curious about its performance in a clinical setting.

A study recently found that ChatGPT was able to accurately answer biomedical and clinical questions on the United States Medical Licensing Examination (USMLE) at a level that approached or exceeded the passing threshold [9]. The study also found that ChatGPT’s accuracy was characterized by high concordance and density of insight, indicating its potential to generate novel insights and assist in medical education [9]. While these results have ignited discussions around potential implications for ChatGPT in healthcare, they also highlight the potential use of this tool in medical education. Whereas the ability of ChatGPT to answer concise, encyclopedic questions have been studied, the quality of its responses to complex medical cases remains unclear [10].

In this study, we aim to evaluate ChatGPT’s performance as a diagnostic tool for complex clinical cases to explore its diagnostic accuracy, the cognitive load of its answers, and the overall relevance of its responses. We aim to understand the potential benefits and limitations of ChatGPT in clinical education.

ChatGPT is powered by Generative Pre-trained Transformer (GPT) 3.5, an LLM trained on a massive dataset of text with over 400 billion words from the internet including books, articles, and websites [8]. However, this dataset is private and therefore lacks transparency as users have no convenient means to validate the accuracy or the source of the information being generated. We plan to conduct qualitative analysis to evaluate the quality of medical information ChatGPT provides.

While ChatGPT is able to generate novel responses that closely resemble natural human language [11] it lacks genuine comprehension of the content it receives or produces.

Once again, this underscores the importance of evaluating the responses provided by ChatGPT. While responses may sound grammatically correct and offer correct medical information, it is essential to assess the overall relevance to the medical question at hand as to not mislead medical trainees.

Medscape Clinical Challenges include complex cases that are designed to challenge the knowledge and diagnostic skills of healthcare professionals [12]. The cases are often based on real-world scenarios and may involve multiple comorbidities, unusual presentations, and diagnostic dilemmas [12]. By employing these challenges, we can evaluate ChatGPT’s ability to answer medical queries, diagnose conditions, and select appropriate treatment plans in a context that closely resembles actual clinical practice [13].

## Materials and methods

### Artificial intelligence

ChatGPT operates as a server-based language model, meaning it cannot access the internet. All responses are generated in real-time, relying on the abstract associations between words ("tokens") within the neural network. This constraint mirrors real-life clinical settings where professionals do not have the freedom to easily access additional scientific literature and also allows us to accurately evaluate ChatGPT’s knowledge.

### Input source

We tested the performance of ChatGPT in answering Medscape Clinical Challenges. These complex cases are designed to challenge the knowledge and diagnostic skills of healthcare professionals [12]. These challenges present a clinical scenario that includes patient history, physical examination findings, and laboratory or imaging results. Healthcare professionals are required to make a diagnosis or choose an appropriate treatment plan using multiple-choice questions [12]. Feedback is provided after each answer with explanations of the correct diagnosis and treatment plan. The distribution of answer options selected by Medscape users is also provided. This feedback mechanism allows an accurate evaluation of ChatGPT’s responses compared to correct answers and also allows us to directly compare its thought process and decision making to healthcare professionals.

Medscape’s Case Challenges were selected because they were open-source and freely accessible. To prevent any possibility of ChatGPT having prior knowledge of the cases, only those authored after its NLP model training in August 2021 were included. This deliberate selection ensures that the chatbot hadn’t been trained on these specific cases beforehand, guaranteeing that each case presented is entirely novel to ChatGPT and that it does not already know the answers.

### Data collection

Data was collected by the three authors, medical trainees (A.H, B.N, and E.T), and all content was reviewed by a Staff Physician (A.K). We felt that it was most appropriate for medical trainees to be the primary evaluator of ChatGPT’s responses, given that it will likely be medical trainees who would rely heaviest on it as an external resource. The three authors (A.H, B.N, and E.T) utilized publicly available clinical case challenges from Medscape, published between September 2021 and January 2023, after the date of ChatGTP’s model 3.5’s training. A total of 150 Medscape cases were analyzed; cases were randomized amongst the three authors with each case being overlapped by at least 2 authors. We excluded any cases with visual assets, such as clinical images, medical photography, and graphs, to ensure the consistency of the input format for ChatGPT.

### Input and prompt standardization

To ensure consistency in the input provided to ChatGPT, the three independent reviewers transformed the case challenge content into one standardized prompt. Each prompt included an unbiased script of what we wanted from the output, followed by the relevant case presentation and multiple-choice answers. The standardization of prompts ensures consistent and reproducible responses across different users and effectively addresses the OpenAI’s placed restriction of using ChatGPT for healthcare advice.

Prompts were standardized as such, all information available on the data extraction supplementary file:

*Prompt 1: I’m writing a literature paper on the accuracy of CGPT of correctly identified a diagnosis from complex, WRITTEN, clinical cases. I will be presenting you a series of medical cases and then presenting you with a multiple choice of what the answer to the medical cases.*

*Prompt 2: Come up with a differential and provide rationale for why this differential makes sense and findings that would cause you to rule out the differential. Here are your multiple choice options to choose from and give me a detailed rationale explaining your answer*.

*[Insert multiple choices]*

*[Insert all Case info]*

*[Insert radiology description]*

### ChatGPT interaction and data extraction

The standardized prompts were input into ChatGPT using the legacy model 3.5, and the model generated responses containing the suggested answer to the case challenge as well as background info on the disease, reasons for ruling in the diagnosis, and reasons for ruling out other diagnoses.

### Primary outcome assessment

All cases were evaluated by at least two independent raters (A.H, B.N or E.T) for each case and blinded to each other’s responses. ChatGTP responses were extracted, and the primary outcome was analyzed based on the percentage of cases for which the answer given was correct.

### Secondary outcome assessment

All cases were evaluated by at least two independent raters (A.H, B.N or E.T). To assess secondary outcomes, we employed three validated medical education evaluation scales:

1. Diagnostic Accuracy: The raters assessed the true positive (TP), false positive (FP), true negative (TN), and false negative (FN) rates of ChatGPT’s answers, considering the suggested differentials and the final diagnosis provided. Each case had four answer options, and ChatGPT’s explanation for each of the four answer options was categorized as either true or false, positive or negative [13]. We then calculated the accuracy, precision, sensitivity and specificity base as shown:
  Accuracy: (TP + TN)/Total Responses
  Precision: TP/ (TP + FP)
  Sensitivity: TP/ (TP + FN)
  Specificity: TN / (TN + FP)
  To further evaluate the model’s performance, we generated a Receiver Operating Characteristic (ROC) curve and calculated the Area Under the Curve (AUC). This involved collecting model scores or probabilities for each instance, sorting instances based on their scores, iterating thresholds to calculate True Positive Rate (TPR) and False Positive Rate (FPR) for each threshold, plotting the FPR against the TPR to create the ROC curve, and computing the AUC to quantify the model’s discriminative ability. This thorough analysis provided both visual representation and scalar measurement to assess the model’s efficacy in diagnostic accuracy.
2. Cognitive Load: The raters evaluated the cognitive load of ChatGPT’s answers as low, moderate, or high, based on the complexity and clarity of the information provided according to the following scale [14]:
  Low cognitive load: The answer is easy to understand and requires minimal cognitive effort to process
  Moderate cognitive load: The answer requires moderate cognitive effort to process
  High cognitive load: The answer is complex and requires significant cognitive effort to process
3. Quality of Medical Information: The raters assessed the quality of the medical information provided by ChatGPT according to the following criteria:
  Complete: The answer includes all relevant information for making an accurate diagnosis
  Incomplete: The answer is missing some relevant information for making an accurate diagnosis
  Relevant: The answer includes information that is directly relevant to the diagnosis
  Irrelevant: The answer includes information that is not directly relevant to the diagnosis
  Using the above scale answers were categorized as one of: complete/relevant, complete/irrelevant, incomplete/relevant, and incomplete/irrelevant [15].
  Discrepancies between raters were resolved through discussion and consensus. In order to assess the inter-rater reliability of our outcomes, we used Cohen’s Kappa coefficient. This statistical measure evaluates the agreement between two raters who each classify items into mutually exclusive categories. It is particularly useful in this study, as it accounts for any agreement that might occur by chance, which is important given the variability of responses from ChatGPT.

### Content analysis

A content analysis was conducted on ChatGPT’s responses to identify patterns of strength and weakness. This analysis focused on the model’s ability to rule out specific differential diagnoses, provide reasonable diagnostic steps, and interpret laboratory values, specialized diagnostic testing, and imaging results. Additionally, we assessed the model’s ability to consider key information relevant to the diagnosis.

### Data analysis

## Results

A total of 150 Medscape cases were included in the analysis (see Table 1), with a total of 600 answer options (four per case) provided to ChatGPT.

**Table 1:** Summary of ChatGPT’s performance on MedScape clinical case challenges.

Case	Case Name	Answer Correct?
1	*Internal Medicine Case Challenge*: *A Teacher’s Assistant With Bipolar Disorder Has Lung Problems*	no
2	*A 27-Year-Old Factory Worker With Incontinence and Imbalance*	yes
3	*Cardio Case Challenge*: *A 17-Year-Old in Cardiac Arrest After Collision Playing Sports*	yes
4	*A 21-Year-Old Man With Epigastric Pain After a Wild Party*	yes
5	*A 19-Year-Old With Hypercholesterolemia*, *Transaminitis*, *and IBD*	yes
6	*Emergency Med Case Challenge*: *A 46-Year-Old Beauty Pageant Winner With Sudden Blindness*	no
7	*Gastro Case Challenge*: *Excruciating Abdominal Pain in a Woman Taking Benzodiazepines and Narcotics*	no
8	*A Teenager Shot Multiple Times Develops Further Complications*	yes
9	*After Consuming Alcohol With Raw Beef*, *a Man Has Seizure*, *Pain*	no
10	*Fingernail*, *Toenail Changes and Flank Pain in a 20-Year-Old*	no
11	*Dermatology Case Challenge*: *Colorful Skin Patches on a Man With Fatigue Who Smokes Cigars*	yes
12	*Diarrhea*, *PPI Use*, *and Pain in a Restaurant Worker From Mexico*	yes
13	*A Woman With AF After Husband’s Death*, *Grandkids’ Drug Abuse*	yes
14	*Emergency Med Case Challenge*: *Hemorrhoids*, *Urinary and Blood Infections in a Woman With Rigors*	yes
15	*Morning Stiffness*, *Dry Eyes*, *Back Pain in a Fit 58-Year-Old*	no
16	*Gastro Case Challenge*: *A Coffee Drinker With Chronic Diarrhea*, *Epigastric Pain*, *and Fever*	yes
17	*Oncology Case Challenge*: *A Construction Worker Who Drinks Daily Has an Eyelid Lesion*	yes
18	*A 22-Year-Old Football Player Who Collapsed Has Urine Changes*	no
19	*A Woman With Multiple New Sexual Partners Has Fatigue*, *Pain*	no
20	*Endo Case Challenge*: *Pubic Hair and Violent Behavior in a Strong 19-Month-Old Girl*	no
21	*A 45-Year-Old Teacher With a Groin Rash That Is Spreading*	yes
22	*Emergency Med Case Challenge*: *Pain*, *Wheezing in a Nonverbal Man Who Keeps Rubbing His Chest*	yes
23	*Recurrent UTIs*, *Ulcerations*, *Foot Drop in 50-Year-Old Woman*	yes
24	*Dermatology Case Challenge*: *Painful Lesions*, *Open Wounds on a 45-Year-Old Woman*	no
25	*Palpitations*, *Cough in a Woman Who Lives Next to a Zookeeper*	yes
26	*Neuro Case Challenge*: *A 35-Year-Old With Angry*, *Aggressive Outbursts*, *Memory Loss*, *and Insomnia*	yes
27	*Gastro Case Challenge*: *Pain*, *Vomiting in a 48-Year-Old on Levothyroxine*, *Metformin*	yes
28	*A Woman Who Owns a Hot Tub and Chickens Has Dyspnea*, *Cough*	yes
29	*Emergency Case Challenge*: *After Argument*, *Unresponsive Woman Found By Her Boyfriend*	no
30	*Beer*, *Aspirin Worsen Nasal Issues in a 35-Year-Old With Asthma*	yes
31	*Endo Case Challenge*: *Amenorrhea for Months*, *Mood Swings*, *Weight Gain in a 38-Year-Old Woman*	yes
32	*Rectal Bleeding in a 47-Year-Old Farmer Who Can’t Pass Flatus*	no
33	*Derm Case Challenge*: *Rash on Chest*, *Buttocks and Toenail Changes in a Middle-Aged Man*	Yes
34	*Gastro Case Challenge*: *A 33-Year-Old Man Who Can’t Swallow His Own Saliva*	yes
35	*A Recently Married 27-Year-Old With Hot Flashes*, *Amenorrhea*	yes
36	*Delirious*, *Incontinent 45-Year-Old Found Crawling on the Floor*	no
37	*Violent Cough*, *Slurred Speech*, *and Ptosis in a Middle-Aged Man*	yes
38	*Emergency Med Case Challenge*: *A 41-Year-Old on Sildenafil With a Headache While Sleeping*	yes
39	*After Unprotected Sex*, *50-Year-Old Has Rash*, *Severe Weakness*	no
40	*Endo Case Challenge*: *Rash*, *Brain Fog*, *and Sleep Issues in a 50-Year-Old IT Director*	yes
41	*Psychiatry Case Challenge*: *Nightmares and Poor Grades in a Third Grader Allergic to Cats*	no
42	*ED Case Challenge*: *After New Sexual Partner*, *Dysuria*, *Discharge in a 21-Year-Old*	no
43	*Loss of Taste*, *Rash*, *and Dyspnea in a 46-Year-Old With GERD*	yes
44	*A 27-Year-Old Woman With Constant Headache Too Tired to Party*	no
45	*Intentional Overdose in a Suicidal 28-Year-Old With Lupus*	no
46	*Oncology Case Challenge*: *A Daily Beer Drinker With Bruises*, *Back Pain*, *and Bleeding*	yes
47	*Neurology Case Challenge*: *A Man With Buttocks Pain*, *Bladder and Bowel Incontinence*	no
48	*A Man With Hypokalemia*, *Sleep Apnea*, *and Resistant Hypertension*	no
49	*An Adopted 43-Year-Old With Bad Breath*, *Dyspnea*, *Dysphagia*	no
50	*Gastro Case Challenge*: *A Daily Cannabis User With Sharp*, *Intense Epigastric Pain*	yes
51	*A 51-Year-Old Man Avoiding Sexual Intercourse Due to Rectal Pain*	yes
52	*Neuro Case Challenge*: *A 16-Year-Old With Quadriparesis After Respiratory Infection*	yes
53	*Psychiatry Case Challenge*: *Alarming Behavior in a 26-Year-Old Soldier and Father of Three*	yes
54	*A Scrotal Rash Lasting Months in a Man With Genital Edema*	no
55	*A 22-Year-Old Female College Athlete With Wild Mood Swings*	yes
56	*Pediatric Case Challenge*: *A 7-Year-Old Boy With a Limp and Obesity Who Fell in the Street*	yes
57	*Endo Case Challenge*: *A Cannabis User With Excessive Sweating and Syncope at Work*	no
58	*An Athletic Teen Suddenly Prone to Falls and Fractures*	yes
59	*An Office Worker With Abdominal Cramps*, *Burning Chest*, *Dyspnea*	yes
60	*Cardio Case Challenge*: *A Confused 35-Year-Old With Headache*, *Fever*, *and Sore Chest*	no
61	*A Woman With Back*, *Chest Pain After Eating Wings at a Restaurant*	no
62	*Neurology Case Challenge*: *A 19-Year-Old With Tinnitus*, *Vision Problems*, *and Headaches*	no
63	*Ob/Gyn Case Challenge*: *A 33-Year-Old Woman Trying to Conceive Has Dyspnea*, *Pain*	no
64	*A Patient Who Collapsed in Agony After Echocardiography*	yes
65	*Oncology Case Challenge*: *A 45-Year-Old Father Seeking Vasectomy Has Alarming Findings*	no
66	*A Divorced Man With Back Pain After Trip With New Girlfriend*	no
67	*Facial Spasms in a Man Recently Released From the Hospital*	no
68	*Gastro Case Challenge*: *A Woman Who Abstains from Alcohol Has Worsening Abdominal Pain*	no
69	*Oncology Case Challenge*: *A Retired Man With Left Upper Quadrant Pain*, *Leukocytosis*	yes
70	*A Coffee Drinker With Sudden-Onset Dyspnea*, *Tachycardia*	yes
71	*PCP Case Challenge*: *Lesions on the Hands*, *Palms*, *and Feet of a 57-Year-Old Man*	yes
72	*A 36-Year-Old Woman With Flatulence and Memory Problems*	yes
73	*A School Nurse With Anxiety*, *Diarrhea*, *Palpitations*, *and Cough*	yes
74	*Cardio Case Challenge*: *Syncope in a 53-Year-Old Woman With Dyspnea and Morning Chest Pain*	yes
75	*A 12-Year-Old With Urinary Retention Who Can’t Grasp Objects*	no
76	*Oncology Case Challenge*: *A 46-Year-Old Mother With Severe*, *Constant Abdominal Pain*	yes
77	*A 42-Year-Old Tennis Player With Dyspnea Blamed on Anxiety*	no
78	*Neurology Case Challenge*: *Drooling and Dysphagia in a Man Who Can’t Speak*	no
79	*Urination Problems After Procedure in a Man Treated for BPH*	yes
80	*Endo Case Challenge*: *A 36-Year-Old Has Cramping*, *Lung Issues and Can’t Lose Weight*	yes
81	*Seizure After Sudden Headache in a 16-Year-Old Cyclist*	no
82	*Cardiology Case Challenge*: *Worsening Chest Pain After a Respiratory Infection in a Man With Hypertension*	no
83	*A 53-Year-Old Waitress With a Cough and Constant Back Pain*	no
84	*Gastro Case Challenge*: *An Incarcerated 24-Year-Old With Dyspnea*, *Fatigue*, *and Chronic Nausea*	yes
85	*17-Year-Old With Hair Loss*, *Dysmenorrhea*, *Thrush*, *and Diarrhea*	no
86	*Sexually Active Man With Foreign-Body Feeling*, *Eye Discharge*	yes
87	*Case Report*: *Cardiac Arrest in a Man Who Has Overdosed*	yes
88	*Primary Care Case Challenge*: *An Accountant With Bilateral Neck Masses*	yes
89	*A 13-Year-Old Athlete With Chest Pain*, *Cough After Practice*	no
90	*Oncology Case Challenge*: *A 37-Year-Old Woman With Multiple Fibroadenomas*	yes
91	*Vaginal Discharge*, *Fever in Pregnant Woman After Hawaii Trip*	yes
92	*Emergency Medicine Case Challenge*: *An Active-Duty Soldier With a Burning*, *Spreading Rash and Sore Throat*	yes
93	*A 42-Year-Old With Declining Cognition and Frequent Vomiting*	yes
94	*Emergency Medicine Case Challenge*: *A Young Girl With Discolored Feet*, *Facial Swelling*, *and Cough*	yes
95	*Endocrinology Case Challenge*: *A 55-Year-Old With Impotence*, *Decreased Libido*, *and Hyponatremia*	yes
96	*5-Month-Old Rushed to the ED for Severe Abdominal Distention*	yes
97	*A 23-Year-Old Unaware She’s Pregnant With Hematuria*, *ECG Abnormalities*	yes
98	*An Anxious Hiker With Recurring Annular Rash and Sleep Loss*	no
99	*Psychiatry Case Challenge*: *A 9-Year-Old With Suicidal Behavior*	no
100	*Neurology Case Challenge*: *Visual and Auditory Hallucinations in a Patient With Parkinson Disease*	no
101	*A 16-Year-Old Girl With Full-Body Rash*, *Dyspnea*, *and Swelling*	yes
102	A Sexually Active 30-Year-Old Woman With Rash and Wrist Pain	yes
103	A Retired Teacher With a Constant Headache and Vomiting	no
104	Star Athlete With a Blinking Fixation Struggling in College	yes
105	Seizures in a 42-Year-Old Who Left a Hospital Against Advice	yes
106	Edible Marijuana Use, Chest Pain, and Cough in a 53-Year-Old	no
107	After Drinking 21 Beers, a 27-Year-Old Can’t Stop Vomiting	yes
108	A Noncompliant Construction Worker With a Pulsating Abdomen	yes
109	A 47-Year-Old With Progressive Dyspnea and Weepy Nodules	no
110	What’s Causing This Rapidly Growing, Golf Ball–Sized Mass?	no
111	Recurrent Syncope in a 30-Year-Old Whose Uncle Died Suddenly	no
112	A Veteran With Lesions, Alcohol Use, and Opioid Dependence	no
113	A Nonverbal 33-Year-Old Woman With Intellectual Impairment	yes
114	A Mail Carrier With Gross Hematuria Whose Sister Has Lupus	no
115	A Sexually Active 23-Year-Old With Seizures and Tongue Pain	no
116	A 17-Year-Old With Hallucinations About Martians and Paranoia	no
117	A 30-Year-Old With a Full-Body Rash, Vomiting, and Confusion	no
118	An Adopted 42-Year-Old With Slurred Speech and Memory Loss	yes
119	A 26-Year-Old With Fever and Malaise Now Can’t Tie His Shoes	yes
120	Decreased Speech and Jerky Eye Movements in a ’Clumsy’ Toddler	yes
121	A 50-Year-Old With Telangiectasia, Cough, and Epistaxis	yes
122	After Travel, a 50-Year-Old Grandfather Has Dyspnea, Fever	yes
123	A 28-Year-Old Soccer Player With Odd Abdominal Pain, Fatigue	yes
124	A 47-Year-Old With Diplopia, Limb Tingling, and Imbalance	no
125	A 47-Year-Old With Diplopia, Limb Tingling, and Imbalance	yes
126	A 53-Year-Old Social Media Worker With Dysphonia and Paresis	no
127	After a Wild Party, a 24-Year-Old Has Intense Abdominal Pain	no
128	An Accountant Who Loves Aerobics With Hiccups and Incoordination	yes
129	A Woman With DVT After a Flight, Anemia, and Bowel Changes	yes
130	A 37-Year-Old Man With Chest Pain and Elbow/Eyelid Papules	no
131	A Marijuana User With Sudden Chest Pain Radiating to His Neck	no
132	A Farmer With Diffuse Pruritus and a Suntan That Won’t Fade	no
133	A 28-Year-Old Writer With Bilious Vomiting After Egg Donation	yes
134	A 35-Year-Old Soldier With Galactorrhea and Amenorrhea	no
135	A 52-Year-Old Man With a Hole in His Jaw and Alcoholism	yes
136	A 57-Year-Old Man With a Fever Who Can’t Stop Bleeding	yes
137	Abdominal Pain, Anemia, and Oliguria in a Distressed Woman	yes
138	A Sexually Active 29-Year-Old Man With a Weak Urine Stream	no
139	A 51-Year-Old Who Lost Her Job Due to Cognitive Decline	yes
140	Strange Stool Color and Fatigue in a Man With COPD and Atrial Fibrillation	yes
141	Painful, Discolored Toes With Sores in a 43-Year-Old Woman	yes
142	Pleural Effusion and an Axillary Mass in a Woman With Hypertension ***(not diagnostic case, cancer origin case)***	yes
143	Penis Injury and Hematuria in a Man Who Fell on a Log	yes
144	A 25-Year-Old Mother With Joint Pain Who Feels Faint	yes
145	A 42-Year-Old Office Assistant With Chronic Leg and Back Pain	yes
146	Blackout at Rest and Slurring in a Man Afraid of COVID-19	yes
147	Chronic Gastritis, a Lesion, and Weight Loss in a Teenager	no
148	Clitoromegaly, Amenorrhea, and Hair Loss in a 32-Year-Old	no
149	A Daily Beer Drinker With Agonizing Gas and Back Pain	no
150	A Former Cocaine User Whose Specialist Told Her She’s Dying	yes

### Primary outcome

**Fig 1:**
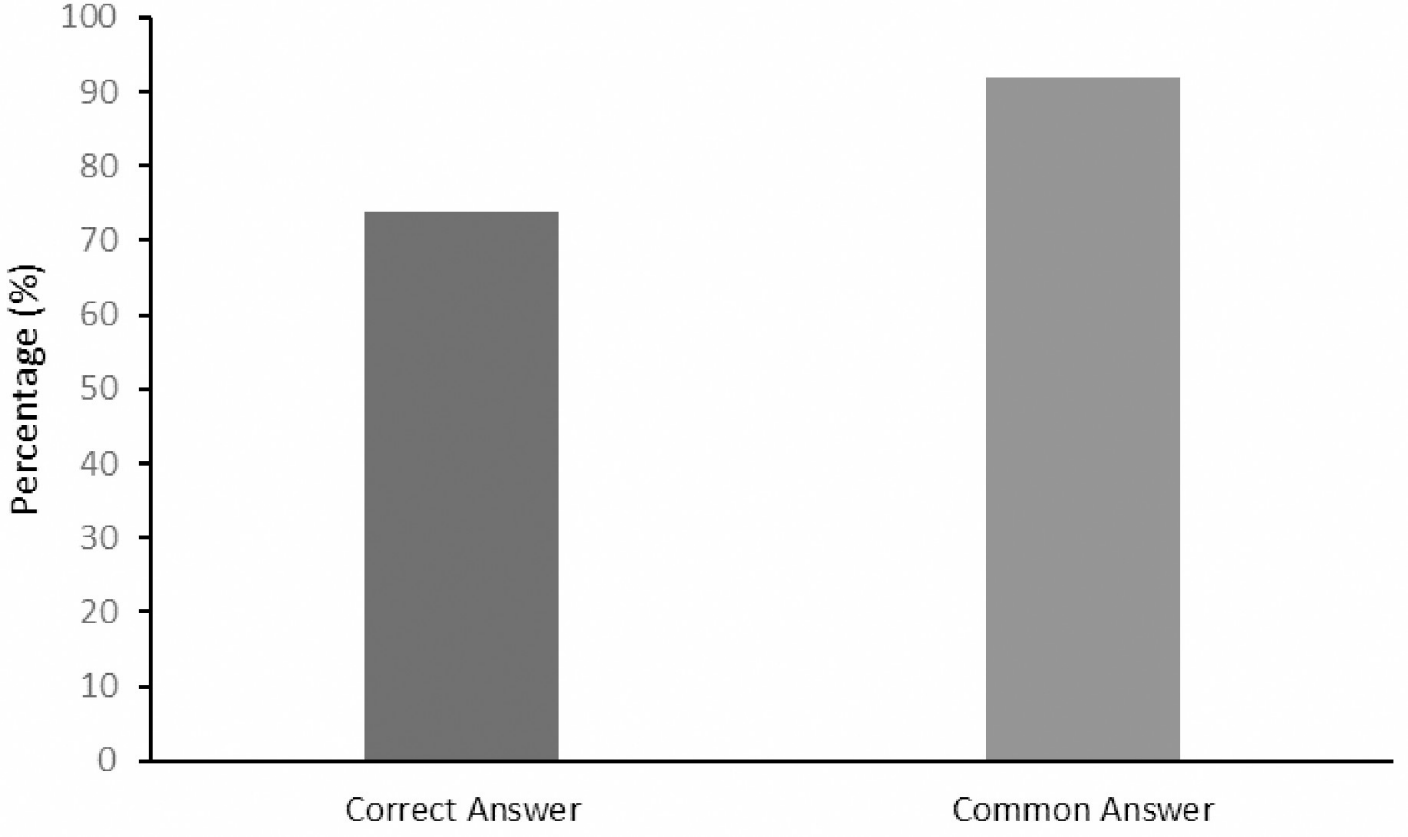
Percentage of correct answers, most common answer and correct answer despite the majority incorrect by ChatGPT 3.5 with MedScape clinical case challenges.

Out of the 150 cases, ChatGPT provided correct answers for 74/150 (49%) of cases (Fig 1). In 92/150 (61%) of cases, ChatGPT provided the answer that the majority of Medscape users provided for the same question.

### Secondary outcomes

#### Diagnostic accuracy

**Fig 2:**
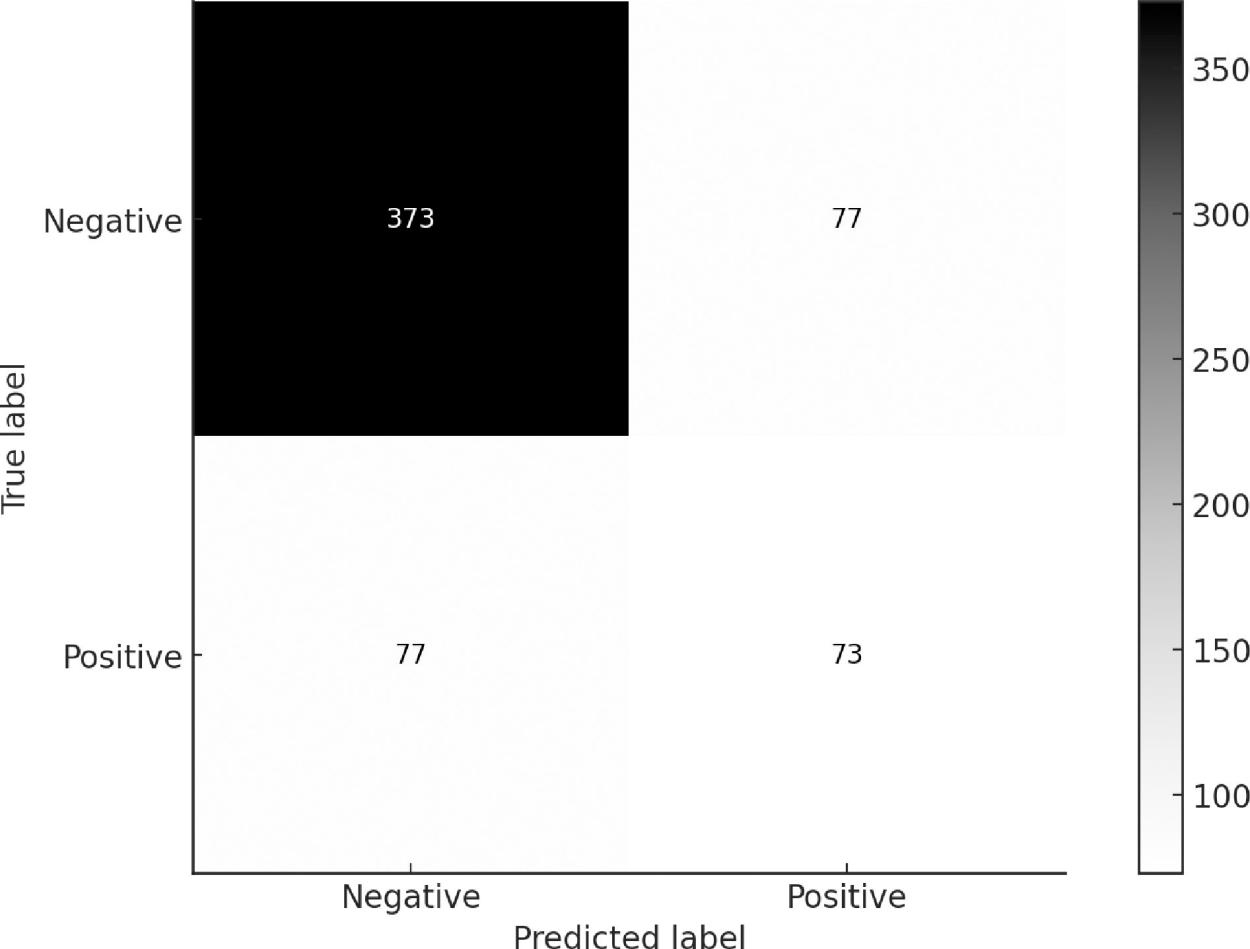
Confusion matrix evaluating the diagnostic accuracy of ChatGPT 3.5, considering each answer within the 150 MedScape clinical case challenges.

There are a total of 150 questions, each with 4 different multiple-choice options, resulting in a total of 600 possible answers, with only one correct answer per question. We found a true positive for 73/600 (12%), false positives for 77/600 (13%), true negatives for 373/600 (62%) and false negatives for 77/600 (13%) (Fig 2). ChatGPT demonstrated an accuracy of 74%, with a precision of 49%. Its sensitivity was 49%, while it achieved a specificity of 83%.The AUC for the ROC was 0.66.

#### Cognitive load

**Fig 3:**
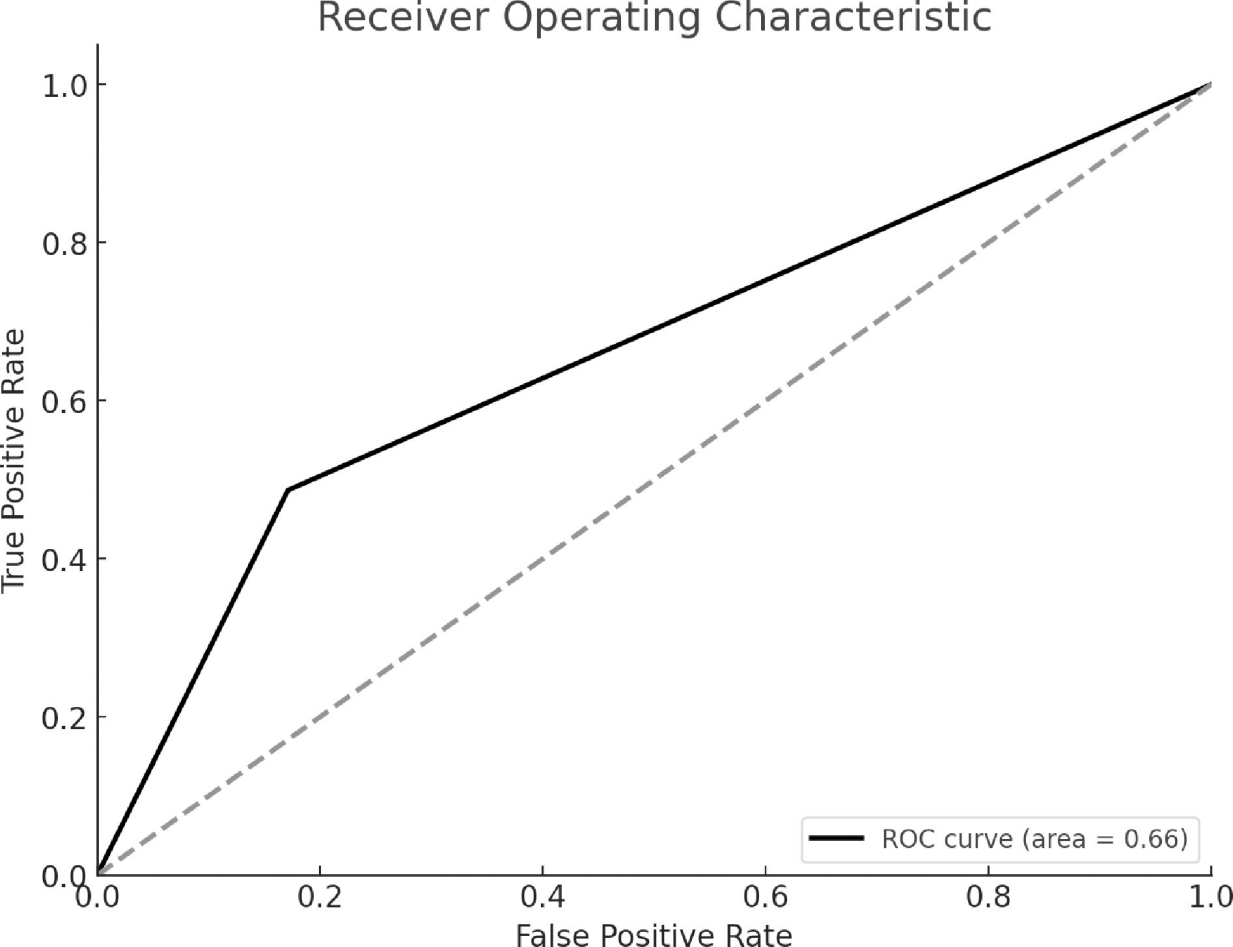
Receiver Operator Curve (ROC) for the diagnostic accuracy of ChatGPT 3.5 answers within 150 MedScape clinical case challenges.

Out of the 150 responses, 78/150 (52%) were categorized as low cognitive load, 61/150 (41%), were found to be a moderate cognitive load, and 11/150 (7%) were classified as high cognitive load (Fig 3).

#### Quality of medical information

**Fig 4:**
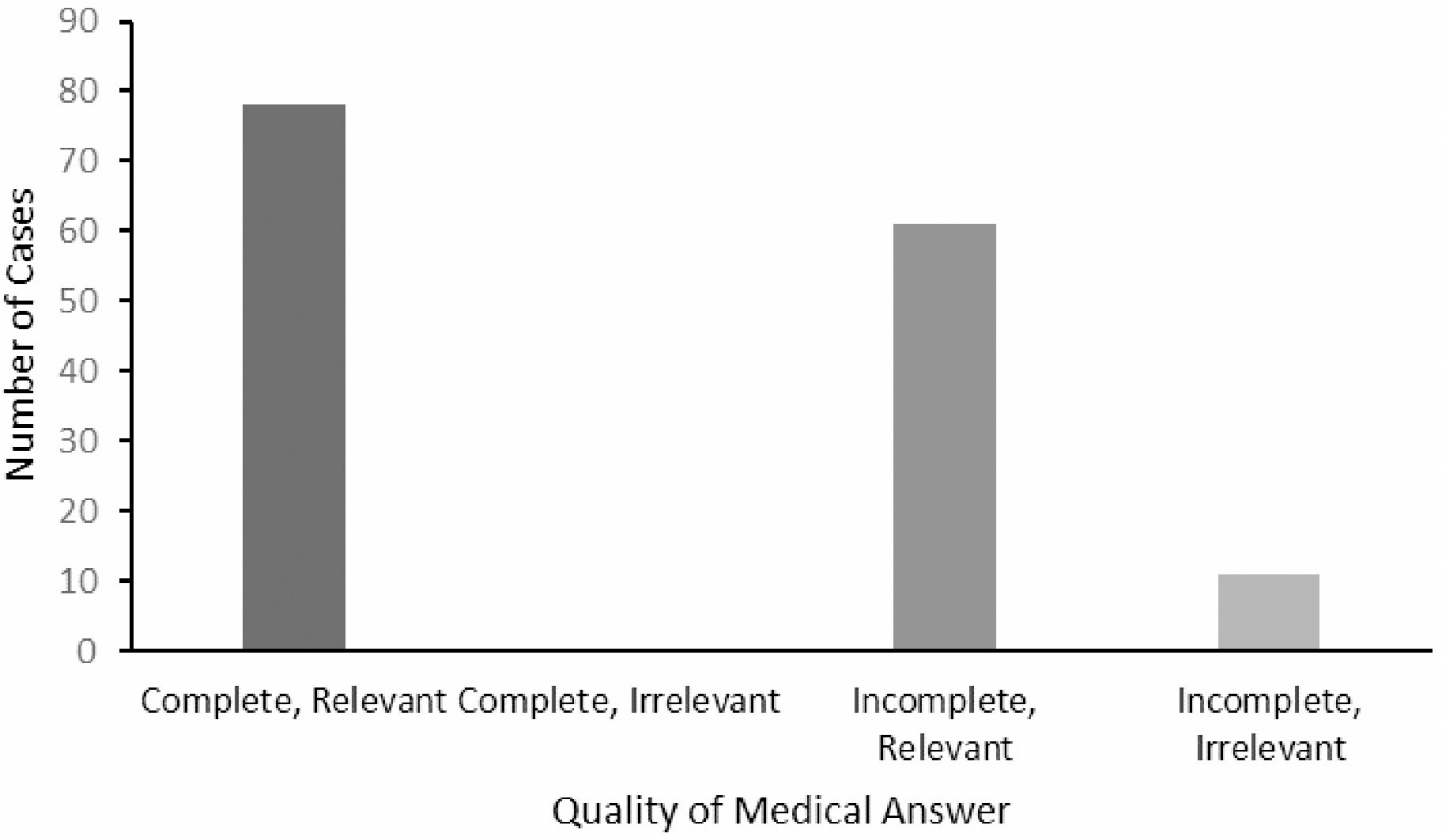
Cognitive load of ChatGPT 3.5 answers given in response to 150 MedScape clinical case challenges.

Responses were complete and relevant for 78/150 (52%) cases. None of the 0/150 (0%) responses were complete but irrelevant, and 64/150 (43%) responses were deemed incomplete yet relevant. Additionally, 8/150 (5%) of the responses were classified as both incomplete and irrelevant (Fig 4).

**Fig 5:**
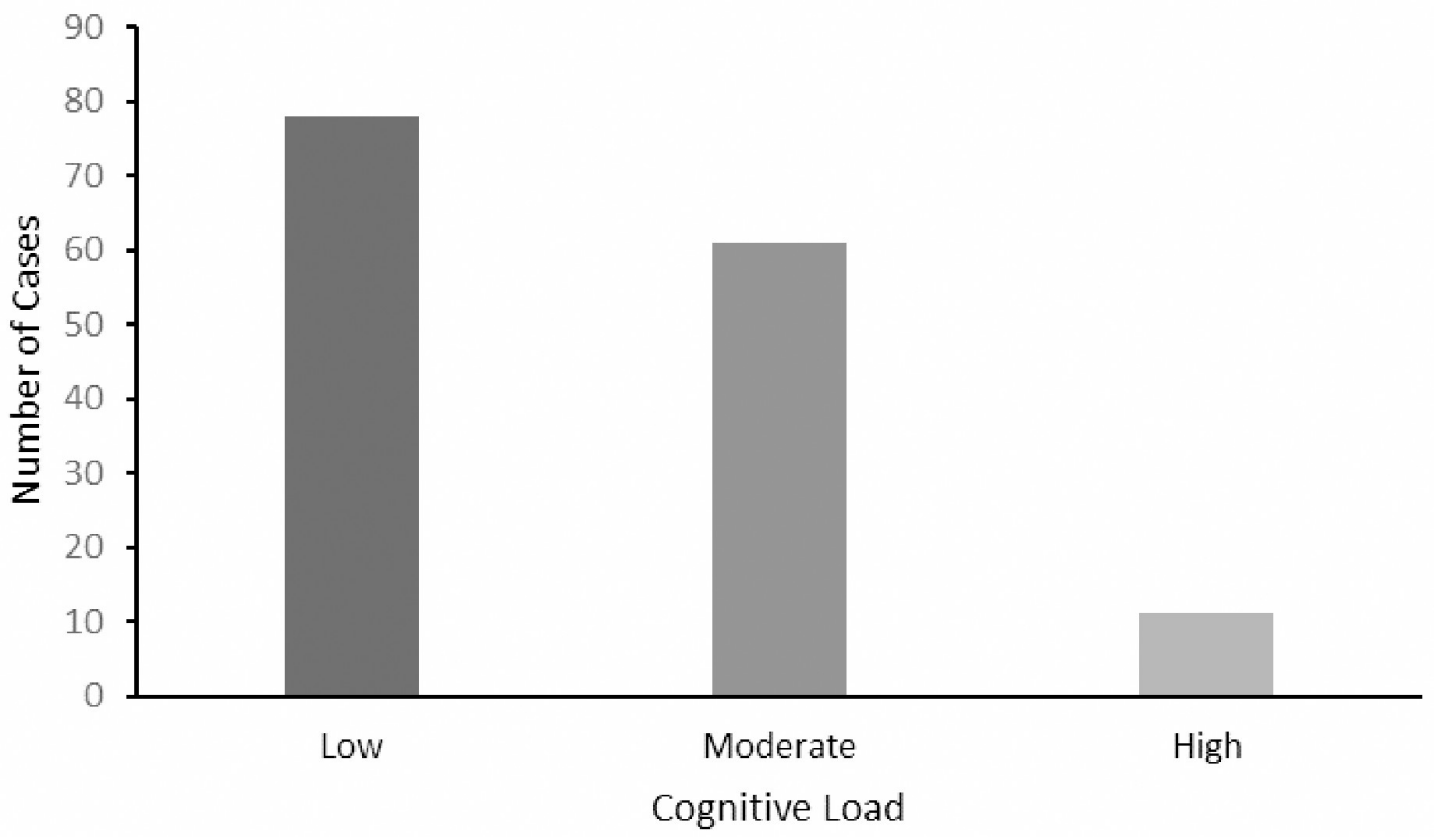
Quality of medical answers given by ChatGPT 3.5 response to 150 MedScape clinical case challenges.

Cohen’s kappa for diagnostic accuracy, cognitive load, and quality of medical information was 0.78 (substantial Inter-rater reliability), 0.64 (substantial Inter-rater reliability), and 1.0 (perfect) respectively (Fig 5).

### Content analysis

We collated the main strengths of ChatGPT’s responses into four themes: clinical rationale, identifying pertinent positives and negatives, ruling our differential diagnoses, and suggesting future investigations. A representative sample of responses including a rationale is provided in Table 2.

**Table 2:** Qualitative analysis of the strengths associated with ChatGPT’s answers in response to MedScape clinical case challenges.

Strengths			
THEME	CASE	GPT Response	Rationale
**Clinical rationale**	A Noncompliant Construction Worker With a Pulsating Abdomen	“The most likely diagnosis in this case is Abdominal Aortic Aneurysm (AAA). The reason for this is due to the physical examination finding of a widened distal circumference of the abdominal aorta, which was confirmed on ultrasound with a diameter of 4.4 cm. The Doppler effect applied to the ultrasound also showed no flow in the area of suspected thrombus or plaque buildup within the vessel, further suggesting an AAA.”	Very consistent in presenting rationale for its findings. Explains the underlying mechanisms of diseases, facilitating education and serving as a refresher on disease manifestations or the rationale behind GPT’s diagnostic analysis.
**Identify pertinent positives and negatives**	Gastro Case Challenge: Excruciating Abdominal Pain in a Woman Taking Benzodiazepines and Narcotics	“The most likely diagnosis for this patient is primary epiploic appendagitis. The patient’s symptoms of severe abdominal pain, nausea, and lack of fever, chills, melena, or hematochezia are all consistent with the presentation of primary epiploic appendagitis. The patient’s history of overweight, sedentary lifestyle, and previous abdominal surgeries, as well as her previous unremarkable CT scans, also support the diagnosis”	Appropriate for identifying pertinent positives and negatives. This provides a fallback option for medical students and clinicians to evaluate even if the final diagnosis is incorrect.
**Ruling out specific differential diagnoses**	Morning Stiffness, Dry Eyes, Back Pain in a Fit 58-Year-Old	“The negative result HLA-B27 is a less supportive finding, as this gene is strongly associated with Ankylosing Spondylitis, but a negative result does not rule out the disease. Additionally, the negative results for rheumatoid factor and anti-CCP antibody levels support a diagnosis of Ankylosing Spondylitis as opposed to a diagnosis of rheumatoid arthritis.”	ChatGPT demonstrated an ability to rule out specific differential diagnoses by justifying its reasons for excluding certain conditions based on the case information provided
**Suggest future investigations**	Cardio Case Challenge: A 17-Year-Old in Cardiac Arrest After Collision Playing Sports	“To rule out other differential diagnosis, I suggest to perform a follow-up ECG, Echo-cardiogram, and Holter monitoring to see if the patient has any underlying heart issue and if there is any arrhythmia or other cardiac event that could be related to the cardiac arrest. Also, a genetic test for Long-QT syndrome is recommended."	Presents missing investigations, and future steps to rule out other diagnoses and to provide more clinical bases to rule in a diagnosis

The models’ main weaknesses were categorized into: misinterpretation of numerical values, inability to handle images, difficulty with nuanced diagnoses, hallucinations, and neglected information (Table 3).

**Table 3:** Qualitative analysis and examples of the weaknesses associated with ChatGPT’s answers in response to MedScape clinical case challenges.

Weaknesses			
THEME	CASE	GPT Response	Rationale
**Misinterprets numerical values**	Gastro Case Challenge: A Coffee Drinker With Chronic Diarrhea, Epigastric Pain, and Fever	“Chronic pancreatitis associated with diverticulosis is ruled out due to the lack of a history of heavy alcohol consumption and normal lipase level.”	ChatGPT may struggle with interpreting numerical values because it relies on the context and language patterns learned during training, which may not always prioritize specific numeric details. As a result, the model might overlook critical numeric information or misinterpret its significance, leading to incorrect conclusions such as incorrectly ruling out chronic pancreatitis, when the patient’s lipase was elevated at 160U/L (Reference range, 10–140 U/L)
**Cannot evaluate imaging**	A 21-Year-Old Man With Epigastric Pain After a Wild Party	“The abnormality seen on the x-ray in the patient is most likely an implanted medical device.”	Without interpretation of the x-ray findings, ChatGPT is very limited in providing the correct diagnosis.
**Difficulty with nuanced diagnoses**	Psychiatry Case Challenge: Nightmares and Poor Grades in a Third Grader Allergic to Cats	“Most likely diagnosis is Generalized Anxiety Disorder. This differential is supported by the patient’s symptoms of frequent waking up in the middle of the night with feelings of breathlessness and fright, difficulty falling asleep on the night before important tests, and tightness in the chest during periods of increased anxiety such as during recess” General Anxiety Disorder resembles Panic Disorder, but the differentiation is that the patient is fearful of the physical symptoms and that they appear “out of the blue”	GPT 3.5 struggles with diseases that have similar yet subtly different presentations. While the patient showed performance-related anxiety and family concerns, generalized anxiety disorder seemed less likely due to their primary symptoms being centred around dysphoric physical symptoms—pointing it to panic disorder. The intricate process of psychiatric diagnosis requires understanding patients and seeing situations through their lens, which ultimately determines their diagnosis.
**Hallucinations**	Edible Marijuana Use, Chest Pain, and Cough in a 53-Year-Old	“The elevated cardiac troponin levels (0.012 ng/mL and 0.016 ng/mL) are diagnostic of AMI, as they indicate myocardial injury.” Ignores history of GERD, weight loss and dysphagia consistent with esophageal adenocarcinoma	ChatGPT occasionally generated incorrect or implausible information. This issue highlights the risk of relying solely on ChatGPT for medical guidance and emphasizes the importance of a human-in-the-loop approach.
**Neglected information**	17-Year-Old With Hair Loss, Dysmenorrhea, Thrush, and Diarrhea	"The most likely diagnosis in this case is Addison’s disease (AD). The patient presents with characteristic symptoms such as chronic oral fungal infections, darkening of skin and oral mucosa, low-grade fever, and diarrhea. Additionally, her physical examination shows evidence of hyperpigmentation in the palmar creases and other areas, which is a common finding in Addison’s disease. The low blood pressure and blood hemoglobin levels support the diagnosis, as Addison’s disease can cause low blood pressure, decreased blood volume, and anemia."	Response excludes a lot of important detail that points towards PGA-1. Findings such as dysmenorrhea and recurrent oral fungal infections were not talked about which are crucial for the diagnosis of PGA-1 gearing away from Addison’s disease. ChatGPT sometimes overlooked key information relevant to the diagnosis.

## Discussion

### Diagnostic accuracy

ChatGPT demonstrated a case accuracy of 49%, an overall accuracy of 74%, a precision of 48.67%,sensitivity of 48.67%,specificity of 82.89%. ChatGPT’s AUC was 0.66, indicating moderate discriminative ability between correct and incorrect diagnoses.

In our assessment of ChatGPT’s diagnostic accuracy in 150 complex clinical cases from MedScape It is important to distinguish between case accuracy and overall accuracy. Case accuracy, which reflects the proportion of cases where the model correctly identified the single correct answer, stood at 49%. However, the overall accuracy, considered the model’s success in correctly rejecting incorrect options across all multiple-choice elements, reached 74.33%. This higher value is due to the ChatGPT’s ability to identify true negatives (incorrect options), which significantly contributes to the overall accuracy, enhancing its utility in eliminating incorrect choices. This difference highlights ChatGPT’s high specificity, indicating its ability to excel at ruling out incorrect diagnoses. However, it needs improvement in precision and sensitivity to reliably identify the correct diagnosis. Precision and sensitivity are crucial for a diagnostic tool because missed diagnoses can lead to significant consequences for patients, such as the lack of necessary treatments or further diagnostic testing, resulting in worse health outcomes.

Overall, these results raise concerns about its accuracy as a diagnostic and education tool for clinicians and medical learners. Several factors led to ChatGPT’s mediocre performance in diagnosing complex clinical cases. Its training data is sourced from diverse texts like books, articles, and websites [8]. These sources offer the AI model a broad understanding of everyday topics and English language nuances but may lack in-depth knowledge in specialized fields like medicine, hindering its ability to diagnose complex cases [16]. Additionally, the training data only includes information up until September 2021 [8]. As a result, recent advancements in various fields may not be reflected in ChatGPT’s knowledge, potentially leading to outdated or inaccurate information being provided by the AI model. To improve diagnostic accuracy, it is crucial that ChatGPT’s training data be augmented with up-to-date, specialized medical information and that the model’s architecture be adapted to handle the nuances of clinical case analysis better.

ChatGPT, provided a considerable number of false positives (13%) and false negatives (13%) which has implications for its use as a diagnostic tool for clinical practice. In the context of false positives and false negatives, it is crucial to consider the role of AI hallucinations as they can significantly impact the accuracy of the information given [17]. Hallucinations refer to outputs generated by an AI model that seem coherent but are not based on factual information, arising from biases, errors, or over-optimization in the model’s training data or its inability to accurately decipher ambiguous or incomplete input data [16]. False positives occur when the AI model incorrectly identifies a condition or disease that is not present, which would lead to unnecessary treatments or interventions that may cause undue stress and anxiety. False negatives occur when an AI model fails to identify a condition or disease that is present, potentially delaying necessary treatments or interventions, and allowing worse outcomes. AI hallucinations contribute partially to the emergence of false positives and false negatives, emphasizing the importance of refining AI models’ training and enhancing their capacity to process intricate information. By doing so, we can potentially improve diagnostic accuracy and reduce the influence of AI hallucinations on medical diagnoses and decision-making processes [17].

### Completeness and relevance of medical answers

ChatGPT’s extensive training in diverse textual data has enabled it to generate complete and coherent responses with proficiency in grammar, context, and a wide range of topics [8]. In most cases, the results produced by ChatGPT are either complete and relevant in 78 out of 150 cases (52%) or incomplete but still relevant, 64/150 (43%), to the user’s inquiry. However, despite its capabilities, ChatGPT may still produce irrelevant responses due to factors such as lack of true understanding, ambiguity or insufficient input, and over-optimization for coherence [16].

Despite ChatGPT’s proficiency in pattern-matching and generating text based on those patterns, its lack of genuine understanding of the content may result in incomplete answers [18]. In some instances, the AI model produces responses that, while syntactically correct and logical in appearance, only partially address the core issue or question. This can be attributed to the model’s struggle to grasp broader context or nuances, such as the interconnectedness of symptoms, patient history, and risk factors. When crucial information is overlooked or relevant details are not connected, the generated answers might be incomplete, not fully meeting the user’s needs or expectations. However, these incomplete answers can still hold some relevance to the topic at hand, providing users with partial information or guidance that could be of value.

In the context of medical learners, ChatGPT’s ability to generate incomplete but still relevant answers can provide valuable insights and learning opportunities. Although the AI model may not always deliver a comprehensive response, the partial information it offers can still contribute to the learner’s understanding of various medical concepts, symptoms, patient histories, or risk factors. These relevant fragments can encourage medical learners to actively engage in critical thinking and problem-solving, prompting them to seek further information to fill in the gaps and develop a more comprehensive understanding of the subject matter. In this way, ChatGPT may hold potential as a supplementary learning tool, however, learners and educators must be wary of the potential for inaccuracy and concepts should be cross-referenced from trusted sources.

In real-world clinical settings, patient information can be ambiguous, incomplete or even incorrect, which poses a challenge for ChatGPT [19]. While patients may not provide all the details relevant to their clinical case, a human healthcare provider can make inferences and use their medical knowledge to put ambiguous details into context, helping them to make informed medical decisions [20]. In contrast, ChatGPT may struggle to make these inferences and as a result, generate irrelevant responses due to an over-reliance on the information provided. As a result, while ChatGPT may assist healthcare providers, it cannot yet replace the expertise and judgment of a human provider [21]. A human healthcare provider can also take into account nonverbal cues and recognize when a patient may omit or miss important details that could affect their diagnosis or treatment [22]. These factors are not easily captured in text-based interactions, making human expertise essential in the diagnostic and treatment process.

### Cognitive load

ChatGPT tends to generate responses with low (77/150) 51%, to moderate (61/150) 41% cognitive load, emphasizing accessibility and readability for users. This characteristic may be advantageous for novice medical students, as it facilitates improved learner engagement and information retention [23]. However, the combination of this ease of understanding with potentially incorrect or irrelevant information can result in misconceptions and a false sense of comprehension. This issue poses a significant challenge for ChatGPT’s application as a medical education tool, as the efficacy of the tool is heavily influenced by the learner’s preexisting knowledge, expertise, and cognitive capacity. In the absence of tailored approaches for these factors, ChatGPT may hinder learners’ ability to apply their knowledge in complex or unfamiliar situations. Addressing this limitation necessitates the development of adaptive algorithms to adjust cognitive load levels based on individual users and the integration of supplementary resources to ensure a comprehensive understanding of the content [24]. Consequently, it is crucial to exercise caution and verify information when relying on ChatGPT for medical inquiries.

### Content analysis strengths and weakness

Our analysis revealed several key limitations in ChatGPT’s diagnostic capabilities. First, the model had difficulty interpreting numerical values, likely due to its reliance on context and language patterns learned during training, which occasionally led to overlooking or misinterpreting critical lab values [9]. ChatGPT’s inability to evaluate laboratory images hindered its diagnostic performance, especially when such images were vital for accurate diagnosis.

ChatGPT also struggled to distinguish between diseases with subtly different presentations and the model also occasionally generated incorrect or implausible information, known as AI hallucinations, emphasizing the risk of sole reliance on ChatGPT for medical guidance and the necessity of human expertise in the diagnostic process [17].

Finally, ChatGPT sometimes ignored key information relevant to the diagnosis. The lack of contextualizing all given information highlights the importance of human input in ensuring critical information is considered during the diagnostic process [21].

### Ethical considerations

As technology becomes increasingly integrated into healthcare, with Electronic Medical Records (EMRs) and other digital tools becoming commonplace, the imperative to securely manage sensitive medical data has never been more critical. Patient privacy and data security are not just ethical imperatives but also crucial for maintaining trust in medical systems [21]. However, as we integrate more advanced technologies into healthcare, new challenges emerge. One significant concern is the potential for algorithms to perpetuate existing biases present in their training data. The selection of this data, often influenced by human biases, can inadvertently reinforce disparities in medical diagnoses and treatment plans, further exacerbating racial and other disparities in healthcare outcomes [25]. Moreover, while AI can provide valuable insights, the importance of human oversight cannot be overstated. Physicians must consider the broader clinical context and individual patient needs, recognizing that the most statistically accurate diagnosis or treatment plan might not align with a patient’s cultural or religious values [21]. As AI’s role in healthcare grows, so does the need for a clear legal framework addressing liability. Questions arise regarding responsibility for misdiagnosis: Should the onus lie with AI development teams, the physicians who rely on these tools, or a combination of both? As we navigate these complexities, the overarching goal remains to ensure that AI serves as a tool to enhance, not replace, the human touch in medicine.

### Limitations

There are several limitations to consider in this study on ChatGPT’s use in medical education. First, our study focused on a single AI model (ChatGTP model 3.5), which may not be representative of other AI models or future iterations of ChatGPT. Our study only utilized Medscape Clinical Challenges, which, while complex and diverse, may not cover all aspects of medical education [12]. The initial approach was to develop a meaningful list of cases that encompasses other aspects of medicine such as management, pharmacotherapy, and pathophysiology, however, the 150 cases from Medscape primarily only focus on differential diagnosis cases [12]. Finally, the input and prompt standardization process relied on the expertise of the authors, and alternative methods of standardization could potentially influence the model’s performance. Future studies should explore different AI models, case sources, and educational contexts to further assess the utility of AI in medical education.

### Future perspectives

ChatGPT has gained significant popularity as a teaching tool in medical education [26]. Its access to extensive medical knowledge combined with its ability to deliver real-time, unique, insightful responses is invaluable. In conjunction with traditional teaching methods, ChatGPT can help students bridge gaps in knowledge and simplify complex concepts by delivering instantaneous and personalized answers to clinical questions [27–31]. However, the use of ChatGPT in medical education poses challenges; outdated databases and hallucinations can lead to the dissemination of inaccurate and misleading information to students [32–35]. To overcome this problem, we foresee future advancements in other LLMs, either trained on medical literature or integrated with real-time medical databases. These specialized models would offer users the advantage of access to accurate medical knowledge and up-to-date clinical guidelines. Beyond its integration, it is important to explore the long-term implications of using LLMs, such as ChatGPT, in health care and medical education. Although numerous studies, including ours, have evaluated ChatGPT for medical education, further research is essential to determine the quality and efficacy of ChatGPT as a tool in this field [32–35].

Future research should focus on discerning the competency of medical professionals who are over-reliant on ChatGPT, assessing patient confidence in AI-supported diagnoses, and evaluating their overall impact on clinical outcomes. These future studies will aid in the development of guidelines for integrating AI into both medical education and clinical practice. While many agree that there’s an urgent need for appropriate guidelines and regulations for the application of ChatGPT in healthcare and medical education, it is equally as important to proceed cautiously, ensuring that LLMs like ChatGPT are implemented in a responsible and ethical manner [26, 36, 37]. As we learn to embrace AI in healthcare, further research in this field will shape the future of patient care and medical training. It’s imperative to proceed with caution, when using ChatGPT as a diagnostic tool and also as a teaching aid and to make sure that it is used in a responsible and ethical manner.

## Conclusion

The combination of high relevance with relatively low accuracy advises against relying on ChatGPT for medical counsel, as it can present important information that may be misleading [24]. While our results indicate that ChatGPT consistently delivers the same information to different users, demonstrating substantial inter-rater reliability, it also reveals the tool’s shortcomings in providing factually correct medical information, as evident by its low diagnostic accuracy. Additional research should focus on enhancing the accuracy and dependability of ChatGPT as a diagnostic instrument. Integrating ChatGPT into medical education and clinical practice necessitates a thorough examination of its educational and clinical limitations. Transparent guidelines should be established for ChatGPT’s clinical usage, and medical students and clinicians should be trained on how to effectively and responsibly employ the tool.

## Supporting information

S1 File (XLSX)
